# Company scaling and its deviations: New indicators for enterprise evaluation and bankruptcy prediction

**DOI:** 10.1371/journal.pone.0287105

**Published:** 2023-10-23

**Authors:** Jing Xu, Xi Chen, Lei Wen, Jiang Zhang

**Affiliations:** 1 School of System science, Beijing Normal University, Beijing, China; 2 Department of Physics, Chalmers University of Technology, Gothenburg, Sweden; 3 Luohan Academy, Hangzhou, China; Beihang University, CHINA

## Abstract

Many studies have shown that scaling laws widely exist in various complex systems, such as living organisms, cities, and online communities. In this research, we found that scaling laws also hold for companies. The macroscopic variables of companies, such as incomes, expenses, or total liability, all have power-law relationships with respect to the sizes of companies, which can be measured by sales, total assets, or the total number of employees. What is more, we also found the power law exponents always deviate from 1. That means large companies naturally have certain advantages, but the widely used financial indicators based on total volume or ratio may not reflect the company’s status well because they are also size-dependent. To tackle this problem, this paper proposes a new set of evaluation indices based on the deviations of the macroscopic variables from the scaling law to eliminate the size-dependent effect. We found that the indicators based on deviations can give more reasonable evaluations for companies and can outperform other conventional indicators to predict the financial distress of companies.

## Introduction

With the continuous development of the global economy, companies play an increasingly critical role in driving forward economic and social development. As the basic unit of the social economy, companies are not only related to the employment and development of individuals but also affect the prosperity and decline of national industries [1]. Companies, as described, are critical connectors between individual employees and entire industries. Thus, our research on companies is an important theoretical problem. What is more, enterprise evaluation has always been an important research area related to credit risk evaluation [2], enterprise merger, acquisition [3], investment, etc.

In the present day, mainstream evaluation indicators typically consist of both financial and non-financial measures. In this paper, we will primarily concentrate on financial indicators, as they can be analyzed quantitatively. Financial indicators can be divided into two types: those based on total volume and those based on ratios. However, through our research, we have found that indicators based on total volume are dependent on the size of a firm, resulting in large firms consistently outperforming small ones. Consequently, it is not possible to compare companies of different sizes using total volume indicators. On the other hand, financial ratio indices are supposed to be consistent across different scales of companies, avoiding the problem of size dependency that total volume indicators face. However, this is not entirely true, as they hold different scaling law parameters.

In order to highlight why those two indicators are size-dependent and what is scaling law, what is the effect of the scaling law, we first need to learn what is the scaling law. Scaling law is a general phenomenon widely existing in complex systems [4]. It describes the systematic macro-level variables that are dependent on the size of the system in a power-law form.

$$\begin{array}{c} Y=\alpha X^{\beta } \end{array}$$ (1)

Where *β* is the scaling exponent, *α* is the normalizing coefficient of the scaling. As an example, one of the most famous scaling laws is the Kleiber′s law, which was found by the biochemist Kleiber in 1932. It dictated that the metabolism of a mammal (*Y*) and its weight (*X*) follow a 3/4 power law relationship which means that the exponent is *β* = 3/4 across different species [5].

Later, many related works found that the scaling laws exist in not only organisms but also social systems, such as cities [6], countries [7], and online communities [8]. For example, Bettencourt et al. found that there are scaling relations between GDP, crime rate, the number of patent applications(*Y*), and urban population(*X*) [9]. And the exponents *β* are all larger than 1, which means the activities with human interactions increase in a faster way than the population when cities grow. We call that super-linear. With the knowledge of super-linear scaling, people can invent a new urban evaluation index based on the scaling laws, which are much better than the conventional widely used per capita indicators as claimed by the paper [9] because the size dependency can be ignored by the new scaling based indicators.

Some papers have also pointed out that the scaling behaviors widely exist in companies such as [10–14]. However, most of the existing studies focus on the perspectives of growth rate but not the macro-level quantities like employees, expenses, etc, and they do not focus on company evaluation. Moreover, limited to the scope of datasets, many existing studies usually did not cover a long study period.

In this paper, we will show that scaling laws of a company exists in almost every financial indicator, and the exponents of the power law relations always deviate 1. This means that the relationships between these financial indicators and company size do not follow the linear hypothesis. Therefore, the ratio indices will change with size. That is to say, the financial performance of large companies will depend on their scale. As a result, they naturally have advantages(super-linear) or disadvantages(sub-linear), depending on whether the exponent is greater or less than 1. Accordingly, we devise a set of new indicators based on the scaling laws to evaluate companies so that the size-dependency of the indicators of total volume and ratios can be eliminated.

The main points of this paper are as follows: First, we give an overall literature review for scaling laws, company evaluations, and bankruptcy, and analyze their strengths and weaknesses based on related works. Secondly, we validate the scaling law of companies using real-world financial data and examine its characteristics across different industries and variables. And discuss the robustness of the scaling law in the S1 File. We also provide an explanation for why ratio and total volume-based indicators fail in evaluating companies. Moreover, we introduce the deviation index and demonstrate its effectiveness through experiments on real-world datasets. These experiments include potential company screening, case studies, and analysis of the Chinese market. We also apply the deviation index to bankruptcy prediction and conduct an industrial-level analysis.

Our contributions are as follows:

1. We reveal the scaling laws of financial variables on companies.
2. We proposed a set of new indicators based on the deviations from the scaling laws (which are abbreviated as deviation indicators) to rank companies.
3. By using the deviation indicators, we can make companies comparisons across industries and time periods, which helps make decisions such as enterprise investment and many other management realistic tasks.
4. Finally, by combining with the classic Cox survival analysis method, we compare our deviation indicators with other well-known total-volume indicators and ratio indicators on company bankruptcy prediction. The result shows that our deviation indicators are more informative on the real situation of company finance and achieved a more accurate prediction result.

## Related works

### Scaling laws in complex systems

In complex systems, one of the most well-known scaling laws is observed in organisms. For example, the size of a fiddler crab’s claws is not linearly or exponentially related to its body length but instead follows a power law relationship. This law can also explain the disproportional growth relationship between various organisms. Specifically, the energy required to sustain growth and survival in a larger organism is not proportional to its growth but instead follows a 3/4 power relationship with body weight (i.e., *E* = *cM*^3/4^). This scaling law, known as Kleiber’s Law [5], referred to as the allometric scaling law, reflects the distinct power coefficients between different variables and scales, indicating different growth rates of various biological variables. There are several theories attempting to explain this pattern, including metabolic theory and life history theory, which suggest that the scales arise from maximizing reproductive energy during natural selection [15].

Interestingly, the idea of non-linear growth has already been extensively explored in urban-scale research. In their papers, Bettencourt et al. have investigated the growth relationship between urban population and specific urban characteristics [9]. The authors have pointed out that the original assumption of urban per capita indicators overlooks the fundamental phenomenon of aggregation [16–21], which arises from non-linear interactions in social dynamics [16, 17, 20] and organization [21, 22] as cities expand.

As a community of people, the online world is also been explored [8, 23, 24]. There are usually two types of scaling laws, one is the super-linear scaling law between the output of the community (such as the number of publications, and the number of posts) and the number of active users; the other is diversity (such as the diversity of labels or words) and the sub-linear scaling law between the number of active users.

Similar relationships exist among companies. Some papers have already pointed out that the scaling behaviors widely exist in companies such as [10–13]. Different from the studies of cities and online communities, in [14], the index “total assets” is selected as the measure of size for companies. Because the power law relations widely exist for any pair of variables, which variable is selected as size doesn’t matter. As to the topic of measuring company size, Coad has extensively discussed this issue [25]. The author has criticized the previous preference for large firms and the inadequacy of research on small companies. Similarly, in the work [26], Santarelli has argued that measures of company size should not be limited to the number of employees or total assets as in the past, and has suggested that other variables such as total sales or assets could also be used as scaling variables in the tables of Chapter 3. This conclusion further demonstrates the flexibility of the scaling rule and provides theoretical support for developing better indicators that are flexible enough to compete with ratio indicators. This allows us to use any type of aggregate indicator as a scale for deviation calculation and evaluation when assessing enterprises.

### Company evaluation

Nowadays, the mainstream evaluating indicators are generally comprised of financial and non-financial indicators. There are wide applications for non-financial indicators based on the famous “Harvard Analysis Framework” [27]. For financial indicators, there are also two types: the indicators based on total volume and the indicators based on ratios. For example, the annual ranking of “The Fortune Global 500”, namely the world’s largest 500 companies, by Fortune magazine is based on the sales revenue of companies, which is a widely used indicator of total volume. However, these indicators are always size-dependent through our research, which means large firms consistently outperform small ones. Therefore, we cannot make cross-size comparisons for different companies by using the indicators of total volume.

Another group of indicators is the financial ratio indices [28–30] which are often used on credit checking for borrowers and loans security guarantee (such as the robustness of a company) in the bank industry [31], and then applied to financial evaluation of companies. The most used financial ratio indices include the current ratio (current assets/current liability), equity ratio (total liabilities/equity interest), and inventory turnover rate (operating cost/average balance of stocks), which measure whether a company has enough capital and solvency. It seems that ratio indices can help avoid the problem of size dependency, which the total volumes indicators confront because they are supposed to be constant across different scales of companies. However, this is not the case because they hold different parameters of the scaling laws.

As to methods in company evaluation, one type of them has put efforts into proposing a complete set of evaluation systems, and how to weigh the final evaluation for company evaluation. Its main goal is to help decision-making [32]. But when mentioning financial performance, many works still use classical indicators like financial total volume indicators or financial ratio indicators. Such as [33] proposed a fuzzy Multiple Criteria Decision-Making (MCDM) model for shipping company performance evaluation. But this work only discusses cases in one industry and does not consider the evaluation across different sectors. For the questions, which industry is more popular at present, and which enterprise in this industry has more potential. Work like this can only answer the latter.

Another way to evaluate a company is to give out a true value for companies, this also be called company valuation. Such as the discounted cash flow methods [34]. [35] discussed its merits and demerits. This method can intuitively provide accurate figures of enterprise value, facilitating comparison. But it is subject to massive assumption bias and even slight changes in the underlying assumptions of analysis can drastically alter the valuation results. What is more, as we discussed above, this approach does not take into account the natural differences in profitability caused by the size differences between large and small enterprises. Therefore, such methods are unfair to small businesses. So we proposed this new indicator to address the problem mentioned before.

### Bankruptcy prediction

Bankruptcy prediction is always a popular research direction which has attracted many researchers to explore a corporate failure prediction model with the best accuracy [36]. Altman first proposed The famous five-factor multivariate discriminant analysis model in 1968, after that, many works focus on the prediction of corporate financial distress. Bankruptcy can be seen as the result of financial distress.

Thanks to the development of statistical techniques and information technology, more and more models have been proposed to solve the problem. Many methods using multiple discriminant analysis (MDA), logit analysis, probit analysis, and neural networks have been used to model bankruptcy prediction problems. Especially since the 1990s, as scholars have become increasingly interested in artificial intelligence technology, neural networks have become one of the most widely used and promising tools.

Also, there are many other models with machine learning methods. Like the rough set theory [37], case-based reasoning [38], support vector machine [39]. Rough set theory has been widely used in various financial decision analysis problems, with reported bankruptcy accuracy ranging from 76% to 88%. Case-based reasoning, as an effective and easy-to-understand method for solving real-world problems, has become an important method in the current field of business fault prediction due to its simplicity, competitive performance with modern methods, and ease of pattern maintenance [40]. Support Vector Machine (SVM), originated in the field of statistical learning theory, was first applied to business failure prediction in 2005 [41], and has been proven to be superior to artificial neural networks [42].

## Materials and methods

### Scaling laws of companies

**Fig 1:**
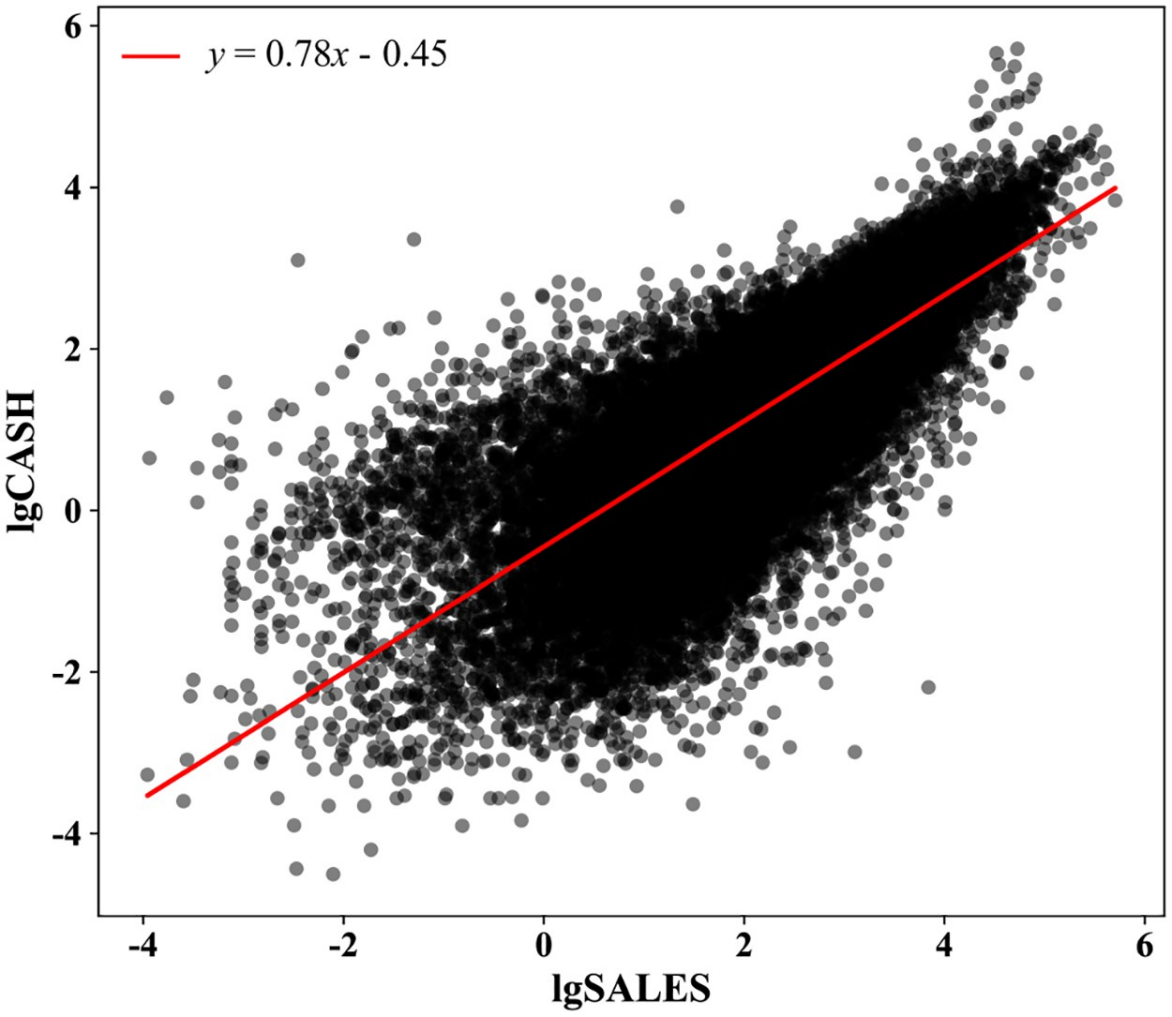
The scaling law of sales and cash for publicly traded companies in the US in 2018. The exponent is 0.78 which means the nonlinear effect on the ratio of cash/sales exists.

Our study finds that the scaling laws, i.e., Eq 1, also hold for companies according to the statistical results of 6645 companies in 2008 publicly traded on the American market. For companies, *X* is the company size, which can be measured as the number of employees, total assets, or sales. *Y* is a total financial indicator such as net income, profit before interest, tax, R&D investment, total liabilities, etc. *β* is the power law exponent, which reflects the relative growth speed of the focal indicator with respect to the company size; and *α* is the scaling normalizing coefficient that reflects the average per unit size value of the focal indicator. Eq 1 holds for various variables across different years [14], as shown in Fig 1.

To estimate *β* and *α* from data, we took the double logarithm of *X* and *Y*, and convert Eq 1 to the following form:

$$\begin{array}{c} \text{lg}\;Y=\beta \;\text{lg}\;X+\text{lg}\;\alpha \end{array}$$ (2)

Then, apply the Ordinary Least Square method to estimate *β* and *α* from the cloud of data (lg *X*_*i*_, lg *Y*_*i*_). There are three situations that can be distinguished according to the values of *β*:

- *β* = 1: The focal financial indicator of the companies increases linearly with size.
- *β* <1: The growth rate of the focal financial indicator is slower than size, which shows a sub-linear relationship.
- *β* >1: The growth rate of the financial indicators is faster than the growth of company size, which shows a super-linear relationship.

Given that the scaling behaviors widely exist for different total financial indicators, let us look at the financial ratio index mentioned earlier, according to Eq 2:

$$\begin{array}{c} ratio=\frac{Y}{X}=\alpha X^{\beta -1} \end{array}$$ (3)

This suggests that a power-law relationship exists between the ratio index $\frac{Y}{X}$ and the company size. However, one may notice that the dependency is weak because the exponent is *β* − 1 which is small. When *β* > 1, the ratio value will decrease with the increase of *X*, and otherwise, if *β* < 1, the ratio value will decrease. When *β* = 1, the ratio is constant across varied sizes.

We systematically study the scaling laws of various financial indicators with respect to sales as shown in Table 1. For discussions of robustness see Fig 9 and Table 12 to Table 14 in S1 (S1 File). We found that *β* < 1 for all of the financial indicators with respect to the size variable of sales. This means that almost all variables will grow slower relative to sales in the year 2018.

**Table 1:** Scaling law between SALES and other variables of the American market in 2018.

Variables	Scaling Coefficient *α*	Scaling Exponent *β* ($)	Standard Deviation of *β*	*R* ^2^	Number of samples
C of SLS	0.81	0.95	0.002	0.89	32976
LT DBT	0.23	0.88	0.006	0.48	24261
TOT LIA	1.51	0.85	0.003	0.75	33182
GR PRFT	0.49	0.83	0.003	0.72	24556
DVC	0.06	0.79	0.006	0.57	13056
INT EXP	0.04	0.79	0.005	0.50	29308
EBITDA	0.26	0.77	0.004	0.50	33306
RET EARN	0.11	0.77	0.008	0.24	31788
ASSETS	5.13	0.76	0.002	0.75	33202
SG&A	0.59	0.75	0.003	0.73	31077
1YR DBT	0.10	0.75	0.007	0.40	19780
CASH	0.35	0.73	0.004	0.53	32252
EMPS	0.01	0.67	0.007	0.35	17838
CMN SH	2.57	0.66	0.004	0.44	33164
TOT TAX	0.08	0.64	0.006	0.31	29949
R&D	0.21	0.55	0.008	0.29	10490
NET INC	0.11	0.54	0.014	0.16	8553

In Table 1, the variables are sorted from top to bottom according to the size of the scaling exponent *β*. This order determines the relative growth rate of the corresponding variables with the company size. Therefore, when the company’s total sales increase, the cost of sales will increase at the fastest rate, long-term debt, total liability, etc.

In this table, we list the statistics of the scaling laws for various variables of Listed Companies of America in 2018, where the scaling exponent is *β*. The standard deviation is calculated by linear fitting of *β*: the smaller the value is, the more accurate the estimate is, thus the result shown in the table is always trustworthy. Scaling coefficient *α* is the coefficient of scaling law, which is interpreted as the value of per-unit sales *Y* in a specific field. *R*^2^ reflects the goodness of linear fitting under the double logarithmic coordinates, and its value is between 0 and 1. The greater the value is, the more the real data conforms to the fitting line, and the more accurate the exponent and coefficient obtained; The sample number is the number of companies used to fit. In addition to the preferred stock dividends and the number of shareholders, most variables show a strong power-law relationship with company size and have high goodness of fit (over 0.50). This can reflect that the variables related to shareholders’ equity and stocks are not only affected by the scale of the company but also accounted for a large part of other factors.

All the above shows different scaling laws among variables, also the scaling laws (Eq 1) hold for different industries are different too. We counted the industry-specific scaling exponents and coefficients in Table 2.

**Table 2:** Different scaling law between cash with respect to sales for different industries in 2018 (sorted by *α*).

Industries	Scaling Coefficient *α*	Scaling Exponent *β* ($)	Standard Deviation of *β*	*R* ^2^	Number of samples
Consumer Discretionary	0.10	0.96	0.009	0.66	5328
Consumer Staples	0.08	0.94	0.014	0.65	2512
Industrials	0.15	0.93	0.008	0.68	6719
Communication Services	0.30	0.87	0.013	0.72	1694
Financials	0.62	0.85	0.014	0.73	1338
Information Technology	0.37	0.85	0.009	0.68	4683
Real Estate	0.54	0.76	0.027	0.58	564
Utilities	0.34	0.74	0.026	0.47	943
Materials	0.44	0.67	0.010	0.48	4079
Energy	0.81	0.62	0.016	0.54	1333
Health Care	1.91	0.55	0.012	0.42	2761

From Table 2, different industries have different exponents of scaling. Among them, Utilities, and Financials are significantly close to 1. That is to say, relationships between financial value and company size in these industries are approximately linear. On the contrary, industries such as Information Technology, Telecommunication Services, and Consumer Discretionary have an exponent of less than 1, which shows these industries may have the phenomenon of scale inefficiency: larger companies have lower per capita sales, lower efficiency, and more severe employee redundancy.

In addition, according to the coefficient *α* in the order of large to small, we can see that the order of per capita sales in different industries sorted from small to large is: Consumer Discretionary <Consumer Staples <Industrials <Communication Services <Financials <Information Technology <Real Estate <Utilities <Materials <Energy <Health Care. The coefficient shows the degree how which one company’s financial value was dependent on its scale. That means for industries such as Energy or Materials, a larger size will cause more cash volume. This result provides a directional reference for decision-makers in these industries to make strategic designations.

### Proposed indicator based on the scaling law

At the beginning of this section, we should introduce our dataset first. We use the COMPUSTATE database from Standard & Poor’s [43]. The data contains financial information for 28,853 North American public companies from 1950 to 2009. Covers all the information in the business income statement and balance sheet.

As previous paragraphs show, scaling laws widely exist in companies, and conventional indicators to evaluate enterprises based on ratios are always size-dependent. Therefore, we propose a new set of indicators for enterprise financial evaluation by taking the scaling laws into account.

The new indicators named deviation indicators are defined based on the deviations of the total financial indicators away from the scaling law (which can be seen as the average behavior of all the companies considered under a given market). For a company *i*, the deviation indicator is defined by:

$$\begin{array}{c} \zeta_{i}=\text{log}(Y_{i})-\text{log}({\hat{Y}}_{i})=\text{log}(Y_{i})-\text{log}(\alpha X_{i}^{\beta }) \end{array}$$ (4)

This indicator can characterize the deviation of *log*(*Y*_*i*_) from the average behavior, i.e., the linear relation $\text{log}({\hat{Y}}_{l})=\text{log}(\alpha X_{i}^{\beta })$, of all the companies in the market. This is also equivalent to a special ratio indicator because $\text{log}(Y_{i})-\text{log}(\alpha X_{i}^{\beta })=\text{log}\frac{Y_{i}}{\alpha X_{i}^{\beta }}$, the ratio between *Y*_*i*_ and $\alpha X_{i}^{\beta }$. This dimensionless ratio indicator is more reasonable than *Y*_*i*_/*X*_*i*_ because size dependency is eliminated completely.

When we take the scaling law (such as the solid red line in Fig 1) as the baseline, the relative position of each point above or below the baseline, that is, the deviation can reflect the success or failure of different companies relative to other companies. Based on this, we can compare any two companies and provide a reliable company ranking (next chapter). Because the scaling law not only exists in all different companies in the market at a specific time but also holds for all time ranges, this deviation indicator can be used to compare companies across different sizes and any time range.

**Fig 2:**
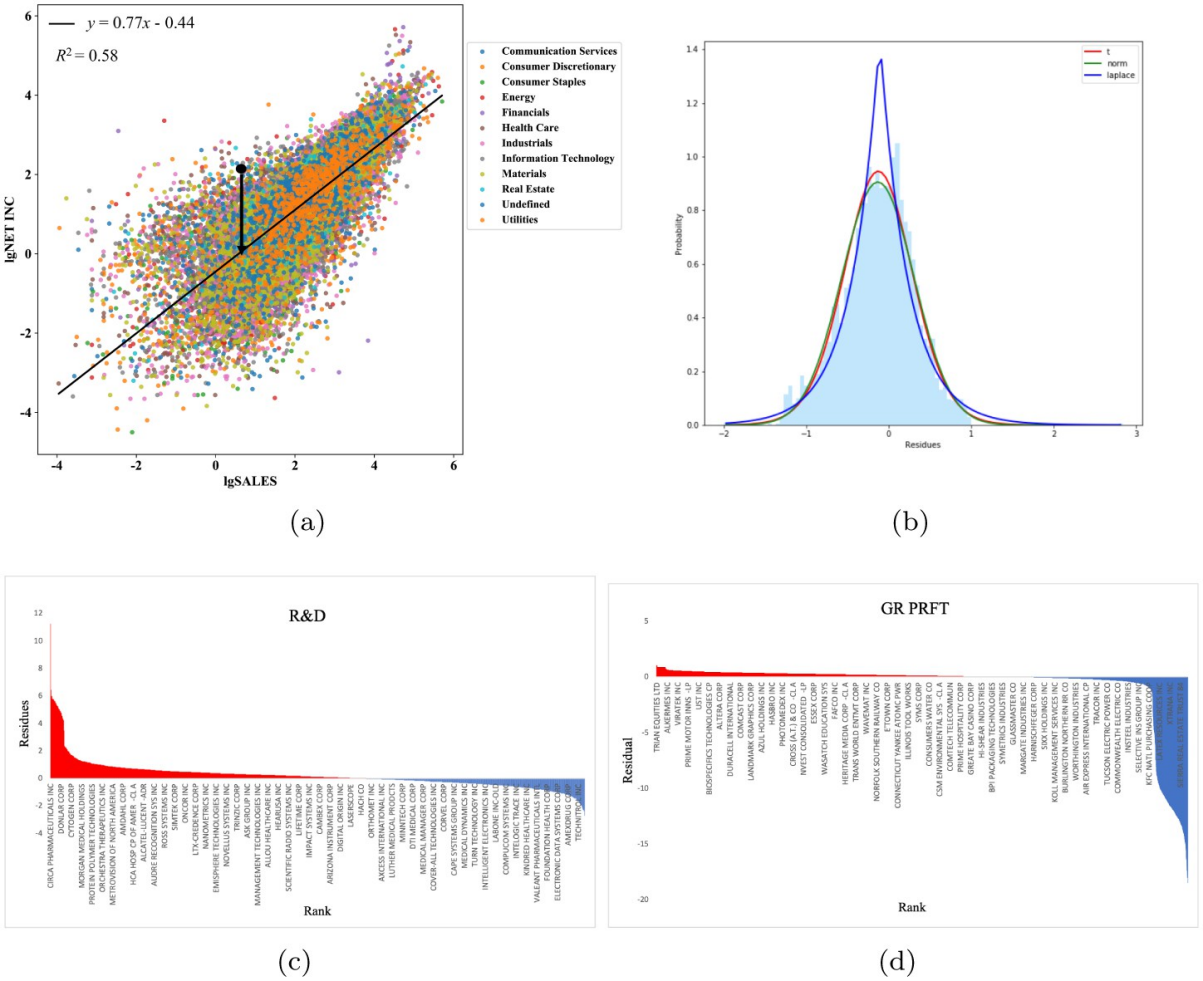
Financial variables exhibit non-linear scaling due to the scaling law of companies.

To illustrate the usefulness of our indicator, we apply the deviation indicator of the scaling law between net income and sales on the Listed Companies in all years from 1950 to 2009 in the COMPUSTAT dataset. The distribution of the indicators and the companies ranking by the deviations are presented in Fig 2.

Where Fig 2(a) shows a typical scaling law (solid line) between Net income and sales through all industries in 2018 (colored dots, different colors represent different economic sectors); the slope of the solid line is *β* = 0.77 (95%CI[0.74, 0.79]), the intercept is ln *α* = -0.44, and the goodness of fit was 58%; Fig 2(b) is a histogram of deviations calculated by Eq 4; the distribution of deviations is well described by a T distribution(red line, the degree of freedom is 8). We also calculated the deviation indicators of (c)R&D and (d)GR PRFT versus sales and listed the top Companies accordingly. All the parameters involved are significant at a 95% confidence level.

From Fig 2, we conclude that in the market, there is a significant power-law relationship between net income and sales. The scaling law can be regarded as a null model of the development of all companies in the market within a time interval. This regularity tells us there is an average level of net income for the given size of sales in the market.

In addition to the relationship between net income and sales, there are similar power-law relationships between other financial indicators and size (not only for sales but also for total assets and the number of employees). However, the regression coefficients of different combinations are different, which shows that the responses of various financial indicators to scale are also different as shown in previous tables. These differences themselves have certain guiding values for the research of companies.

Furthermore, the distribution of deviation also shows obvious peak fat tail characteristics, which is a common phenomenon in the financial market, especially in the distribution of financial asset returns, such as stocks, bonds, and so on. At the same variance level, compared with the normal distribution, the probability of frequent events is weakened by the fat tail, which is prone to many extreme cases. This is also the reason for the uneven development of companies in the market and the coexistence of companies far above and far below the average. There is no phenomenon that most companies concentrate on floating up and down the level. It is worth noting that the mean value of company deviation in different index combinations is around 0, which further verifies the rationality of using scaling law as a null model to measure the level of market development.

## Results

### Ranking potential companies with deviation indicators

In this section, we will first show how to rank companies using deviation and financial ratio metrics. We compare the conventional ratio index and the deviation indicator between gross profit and sales on a set of the same companies, and the top ten companies are listed respectively as shown in Table 3 (the values are sorted from large to small).

**Table 3:** GR PRFT_ SALES(The deviation *ζ* obtained when *X* takes SALES and *Y* takes GR PRFT, in Eq 4) vs. Profit ratio(GR PRFT / SALES).

Screening by deviation indicator			Screening by financial ratio		
Name	Sectors	Life span	Name	Sectors	Life span
TRIAN EQUITIES LTD	Financials	1985–1995 No longer files with SEC among other possible reason	TRINIDAD CORP	Consumer Discretionary	1983–1996 No longer files with SEC among other possible reason
HYDROGEN POWER INC	Materials	1984–2007	MIDWEST REALTY & FINANCE INC	Financials	1974–1994 No longer files with SEC among other possible reason
CONGRESS STREET PPTYS INC	Financials	1987 IPO-1995 Acquisition or merger	NEW WORLD BRANDS INC	Consumer Discretionary	1987 IPO-2010
COMCAST CABLEVISION -PHILA	Financials	1986 IPO-1994 Acquisition or merger.	ADVANCED PHOTONIX INC -CL A	Information Technology	1991 IPO-2014
TRINIDAD CORP	Consumer Discretionary	1983–1996 No longer files with SEC among other possible reason	ATLANTIC INDUSTRIES INC	Consumer Discretionary	1981–2000 No report.
MIDWEST REALTY & FINANCE INC	Financials	1974–1994 No longer files with SEC among other possible reason	TECFIN CORP	Financials	1982–2000 No report. But pricing continues.
NEW WORLD BRANDS INC	Consumer Discretionary	1987 IPO-2010	HEALTH ADVANCEMENT SVCS INC	Health Care	1990–1997 No report.
ADVANCED PHOTONIX INC -CL A	Information Technology	1991 IPO-2014	TRAVLANG INC	Information Technology	1984 IPO-2011 No report.
ATLANTIC INDUSTRIES INC	Consumer Discretionary	1981–2000 No report.	NORTON DRILLING SERVICES INC	Energy	1987 IPO-1999 Acquisition or merger
GLOBUS GROWTH GROUP	Financials	1980–2011 Now a private company	KRAUSES FURNITURE INC	Consumer Discretionary	1986–2011 No report. But pricing continues.

It should be noted that from Eqs 3 and 4, it can be seen that when the corresponding value is 0, neither of them can be calculated. It can be clearly seen from the table that 5 of the top ten companies screened by the two are overlapped but ranked differently. The companies screened by the deviation indicator are more likely to survive longer or to be acquired (acquisition or merger).

**Fig 3:**
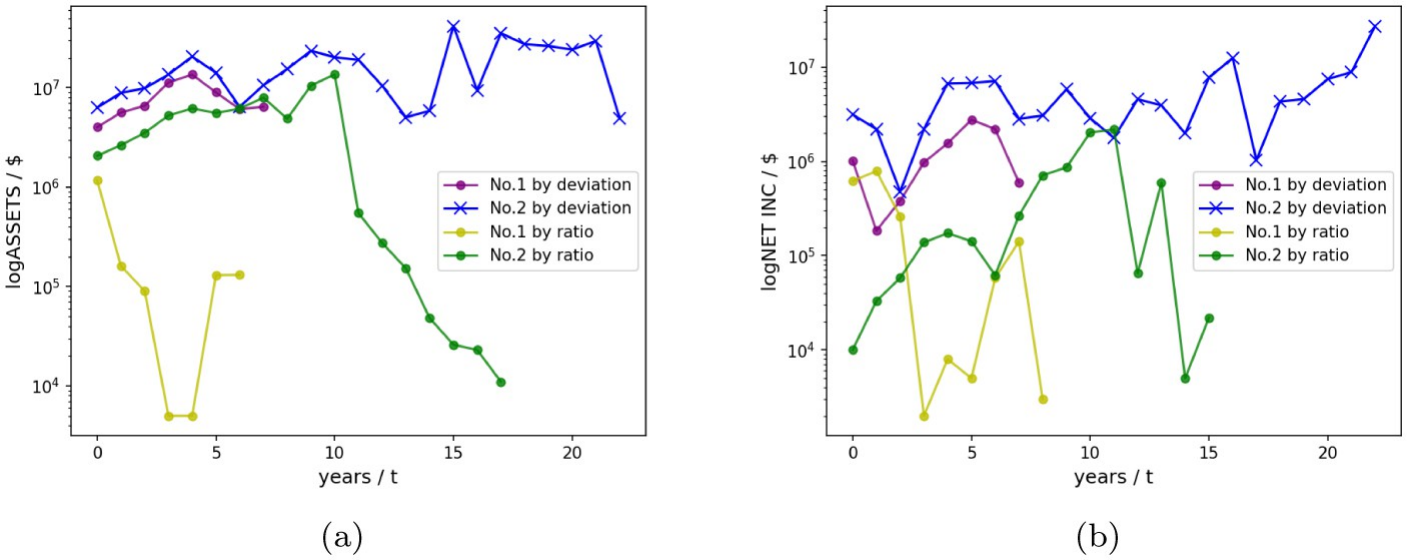
Comparison of original data(The figure is plotted after removing the data zero and taking the absolute value. Represents the time series data of several companies from the first year of listing to the deadline of the database).

In order to further confirm the deviation of the ability to filter for the steady development of companies, we visualized the total assets (asset size) and net income (profitability) of the first two companies on a logarithmic scale from the level of real data on corporate development shown in Fig 3.

From Fig 3, we observe that the companies selected by the deviation indicator have more stable development than the ones selected by the ratio indicator, although they have similar asset sizes at the beginning.

Similarly, we select the compare indicators and list the top 10 companies from the perspective of operating cost (SG&A) and sales as shown in Table 4.

**Table 4:** SG&A_SALES deviation(The deviation *ζ* obtained when *X* takes SALES and *Y* takes SG&A, in Eq 4) vs. SG&A/SALES.

Screening by deviation indicator			Screening by financial ratio		
Name	Sectors	Life span	Name	Sectors	Life span
CIRCA PHARMACEUTICALS INC	Health Care	1975–1995 Acquisition or merger.	CIRCA PHARMACEUTICALS INC	Health Care	1975–1995 Acquisition or merger.
HYDROGEN POWER INC	Materials	1984–2005 Still alive.	HYDROGEN POWER INC	Materials	1984–2005 Still alive.
WESTMINSTER CAPITAL INC	Consumer Discretionary	1979–2001 Acquisition or merger.	WESTMINSTER CAPITAL INC	Consumer Discretionary	1979–2001 Acquisition or merger.
BIOGEN IDEC INC	Health Care	1991IPO Still alive.	INNOVATIVE SHIPPING SYS INC	Industrials	1986–1997 No report.
GILEAD SCIENCES INC	Health Care	1992 Still alive.	GEOWASTE INC	Industrials	1974–1998 Acquisition or merger.
INDEVUS PHARMACEUTICALS INC	Health Care	1990IPO-2011 Acquisition or merger.	SPORT OF KINGS INC	Consumer Discretionary	1983–1996 No report. But pricing continues.
MEDIMMUNE INC	Health Care	1991IPO-2011 Acquisition or merger.	INDEVUS PHARMACEUTICALS INC	Health Care	1990IPO-2011 Acquisition or merger.
EMBREX INC	Health Care	1991IPO-2011 Acquisition or merger.	LML PAYMENT SYSTEMS INC	Information Technology	1985–2009 Still alive.
SEPRACOR INC	Health Care	1991IPO-2011 Acquisition or merger.	EMBREX INC	Health Care	1991IPO-2011 Acquisition or merger.
VERTEX PHARMACEUTICALS INC	Financials	1991IPO Still alive.	QUANTRX BIOMEDICAL CP	Health Care	1988 IPO-2009 Still alive

One may notice that the top three companies selected by the two indicators are exactly the same, but more companies selected by the deviation indicator have survived or been acquired.

Table 4 also shows that many selected companies are from the Health Care industry which means for the US market in 1990, the operating cost of the Health Care industry was significantly higher than that of other industries. This reminds us that when we comprehensively compare two companies, we should notice if they are in the industry. If they are not, we need to study their relative developments in their own industry.

### Multi-variate evaluation

In the previous paragraphs, we compared deviation indicators and ratio indicators within a single pair of variables and achieved better performance. However, a comprehensive evaluation of a company requires comparing a set of variables. Thereafter, a method of multi-variate evaluation based on deviation indicators is needed.

In order to conduct a multivariate investigation of the results of the two types of index screening, in this Section, we select the first example of the two types of index rankings and use the same method of calculating *r* in formula 5 for analyzing multiple variables.

$$\begin{array}{c} r=1-cdf(\zeta ) \end{array}$$ (5)

Among them, *cdf*(*x*) ≡ *Pr*(*ζ* > *x*), represents the cumulative distribution function of the deviations (*ζ*) of all companies in the industry in that year. The ranking defined in this way indicates the top percentage of the focused company according to its deviation value relative to other companies.

Next, we applied this new index to representative companies, TRIAN EQUITIES LTD and TRINIDAD CORP, which are the top two companies sorted by deviation indicator within the financial industry, in 1990. We select company data in 1990 as the research object cause it is one of the beginning years of our dataset and also the beginning year of these selected companies. Other years can also be chosen to conduct comparisons if one needs to know its relative development in that year. We select sales as the size proxy for companies and calculate their ranking *r* for seven representative variables. The results are shown in Table 5.

**Table 5:** Results of in industry assessment in 1990.

Financial Index	TRINIDAD CORP		TRIAN EQUITIES LTD	
	*ζ*	*r*	*ζ*	*r*
ASSETS	0.14	27.39%	5.91	0.23%
EMPS	1.73	0.30%	4.64	0.34%
CASH	1.60	1.64%	3.47	0.68%
GR PRFT	0.41	2.94%	7.66	0.05%(NO.1)
NET INC	-6.28	100%	8.08	0.11%
C of SLS	-3.18	100%	6.37	0.23%
TOT LIA	1.40	0.59%	6.33	0.45%

**Fig 4:**
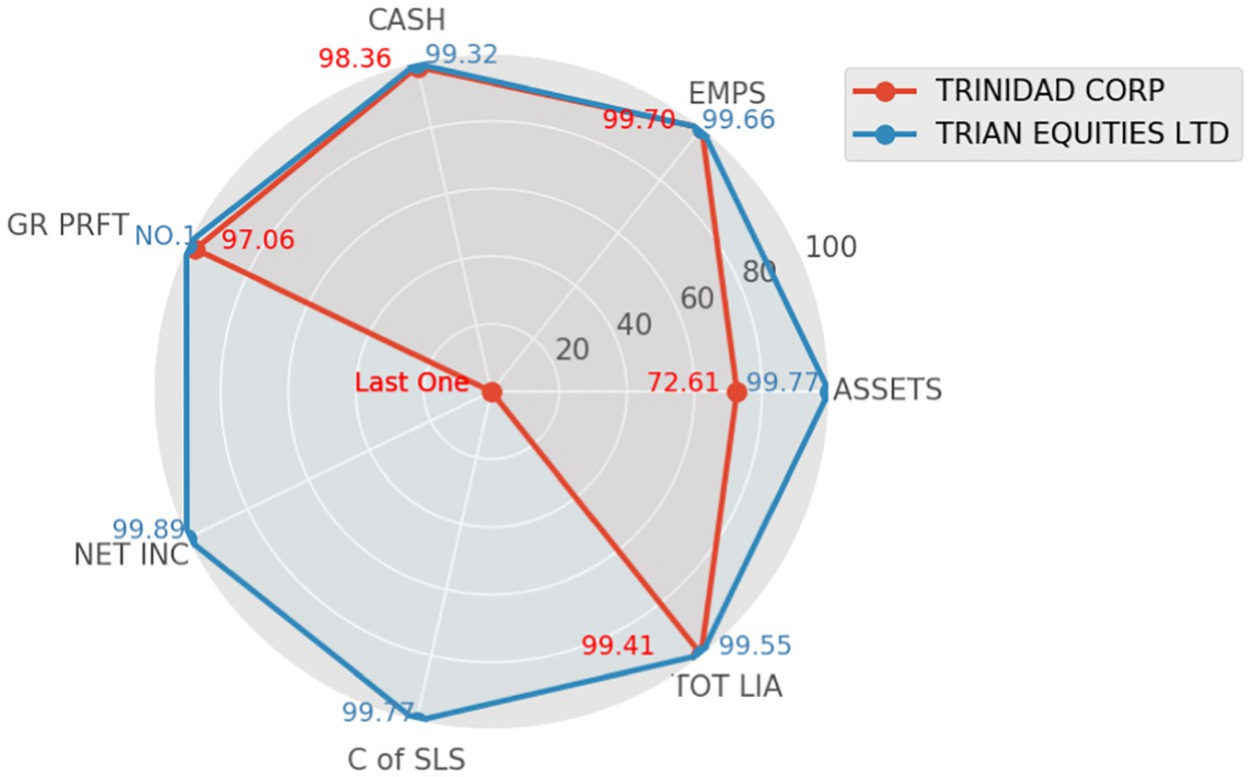
Ranking comparison radar chart (1990).

The Radar chart in Fig 4 intuitively shows the comparison of the two companies in multiple dimensions. The red and blue lines respectively indicate the evaluation performance of various aspects of the company screened by the ratio and deviation indicators. *ζ* represents the specific deviation value of the corresponding company, and *r* represents the ranking of the company in all aspects of the corresponding industry (the top percent). The radius of the radar chart represents 1 − *r*. The larger the radius, the better the performance in this aspect, and vice versa.

According to the radar chart and Table 5, it is noticeable that TRIAN EQUITIES LTD company performs better, and TRINIDAD CORP performs similarly on features of net profit (PROFT), cash (CASH), employees (EMPS), and total liability (TOT LIA) as TRIAN EQUITIES LTD, but worse on features of net incomes (NET INC) and cost of sales (C of SLS) because its deviation value is a large negative number. It can be seen that the overall performance of TRINIDAD CORP is slightly lower than that of TRIAN EQUITIES LTD.

To compare the methods, we also show a comparison of these two selected companies on the same seven features but with a ratio indicator as shown in Table 6. Although TRIAN EQUITIES LTD can still be better, the overall performance of TRINIDAD CORP appears to be very poor because of the two negative properties with large absolute values (NET INC/GR PRFT, and EBITDA/GR PRFT). If the smaller the indicator ratio the worse is the company, then TRINIDAD CORP seems very poor. But this is unreasonable because several reasons can lead to a negative ratio. For example, the negative signs that appear may be due to either the denominator or the numerator; or all companies in the sector may be negative, etc. In addition, we also observe that the ratio of TOT LIA/ASSETS is abnormally large. This reflects the sensitivity of the ratio indicators. Therefore, ratios always have large fluctuations, which interferes with us comparing different companies.

**Table 6:** More financial ratio indicators of the two companies.

1990	GR PRFT/SALES	NET INC/GR PRFT	EBITDA/GR PRFT	TOT LIA/ASSETS
TRINIDAD CORP	1	-8	-7	7
TRIAN EQUITIES LTD	1.080	0.540	1	0.093

To obtain a comprehensive judgment, we can average all ratio values for all properties, then TRINIDAD CORP will have an unreasonable small ratio: -7. However, if we average *r* for all variables of the same company, we obtain a reasonable evaluation: 33.27%. In summary, the deviation indicator is better in this comparison.

### Deviation analysis on Chinese market

To further prove that scaling laws widely exist in financial systems not only in North America. So here we expand the analysis above in the Chinese market. Data provided by the Wind dataset [44] with 3073 companies of 12 sectors during 1996–2020.

**Fig 5:**
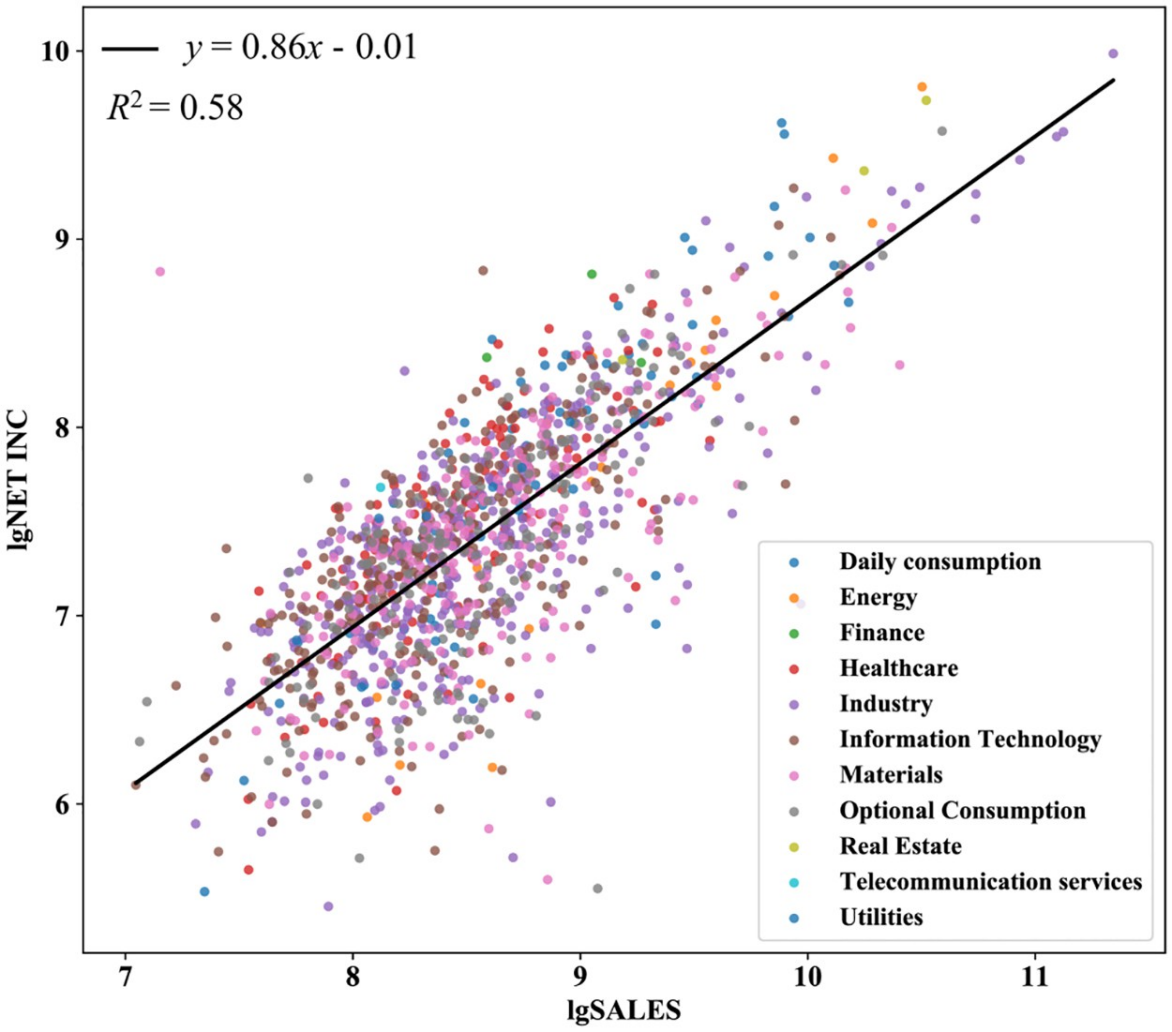
Scaling law of Chinese market between net income and total sales in 2020.

As shown in Fig 5, scaling law also makes sense in the Chinese market. Scaling laws for other variables see S1 File. Since this, we can use this in the evaluation of companies. For example, we choose the deviation of net income to filter companies with higher incomes in 2020. In the same way, the top 10 is shown in the following Table 7.

**Table 7:** NET INC_SALES deviation(The deviation *ζ* obtained when *X* takes SALES and *Y* takes NET INC, in Eq 4) vs. NET INC/SALES. The unit is converted into US dollars.

Screening by deviation indicator			Screening by financial ratio		
Name	Sectors	Life span	Name	Sectors	Life span
Jilin Liyuan Precision Manufacturing Co., Ltd.	Materials	2007–2020	Jilin Liyuan Precision Manufacturing Co., Ltd.	Materials	2007–2020
Kunlun Tech Co., Ltd.	Information Technology	2015–2020	Kunlun Tech Co., Ltd.	Information Technology	2015–2020
Ningbo Donly Co., Ltd	Industry	2004–2020	Ningbo Donly Co., Ltd	Industry	2004–2020
Muyuan Foods Co., Ltd.	Daily consumption	2014–2020	Jiangsu Nanfang Bearing Co., Ltd.	Optional Consumption	2011–2020
Haoxiangni Health Food Co., Ltd.	Daily consumption	2011–2020	Shenzhen Shengxunda Technology Co., Ltd	Information Technology	2016–2020
China Yangtze Power Co., Ltd.	Utilities	2002–2020	Haoxiangni Health Food Co., Ltd.	Daily consumption	2011–2020
East Money Information Co., Ltd.	Financials	2006–2020	Hangzhou Tigermed Consulting Co., Ltd.	Health Care	2012–2020
Jiangsu Nanfang Bearing Co., Ltd.	Optional Consumption	2011–2020	Hithink Royalflush Information Network Co., Ltd.	Financials	2006–2020
Hangzhou Tigermed Consulting Co., Ltd.	Health Care	2012–2020	East Money Information Co., Ltd.	Financials	2006–2020
Hithink Royalflush Information Network Co., Ltd.	Financials	2006–2020	Shandong Sunway Chemical Group Co., Ltd.	Industry	2007–2020

Since most of the records included in the wind database are large listed companies, there are very few bankrupt and dead companies. Therefore, from the perspective of survival or not, it is not very clear what the differences are between the companies selected by the two indexes. Secondly, it can be seen that for the Chinese market in 2020, from the perspective of enterprise names included in the top ten, most of them are consistent. The top three are completely consistent, and 5 of the other 7 companies are consistent but have different rankings. Specifically, the fifth and seventh to tenth places selected from the deviation index are the sixth, ninth, fourth, seventh, and eighth places in the ratio index. This does not seem to indicate any significant differences directly from the table. So we also compared the real-time series data of some enterprises with the same ranking. Since the top three companies in Table 7 are the same, so we compare the real-time series of companies in 4 to 5 lines.

It can be seen that companies selected by deviation tend to be more stable, and there has been steady growth in terms of assets and cash flow for at least recent five years. To furtherly explore, we conduct a multi-variable evaluation of the company Muyuan Foods Co., Ltd. and Jiangsu Nanfang Bearing Co., Ltd. as mentioned before in their corresponding industry.

**Fig 6:**
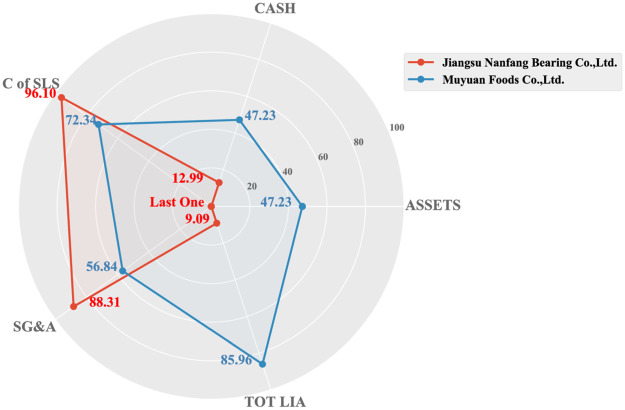
Ranking comparison radar chart of the Chinese market (2020).

**Fig 7:**
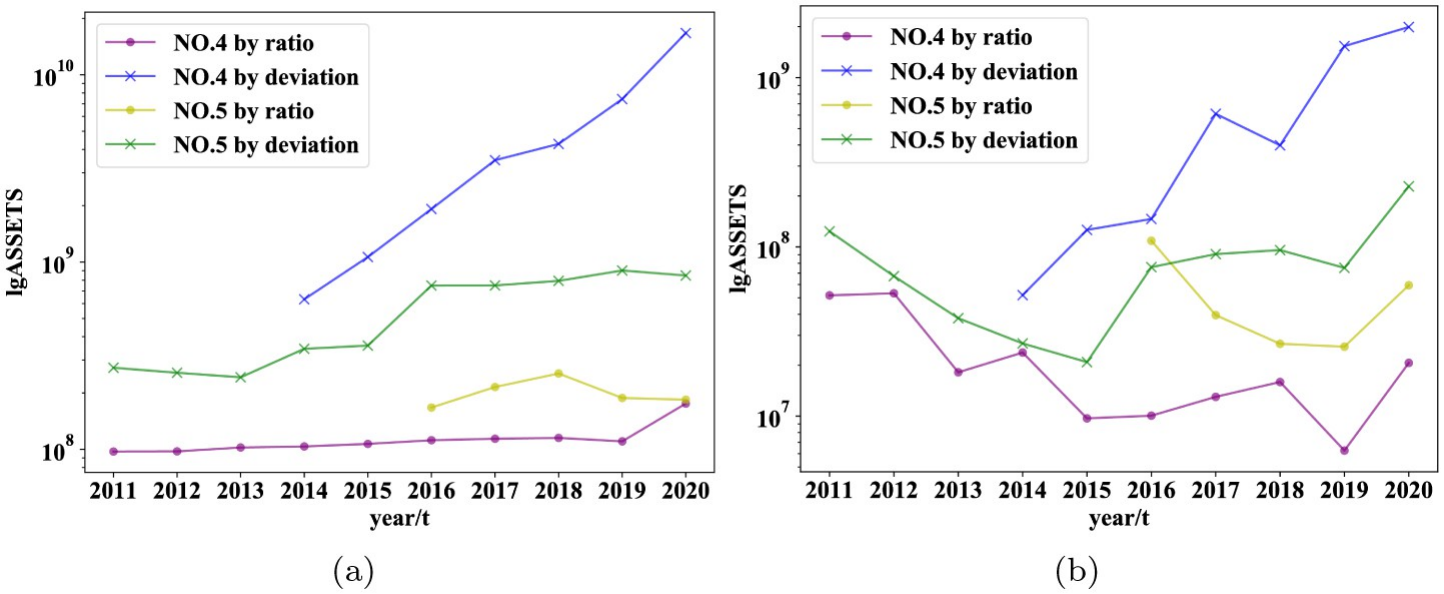
Comparison of original data. (The figure is also plotted after removing the data zero and taking the absolute value. Represents the time series data of several companies from the first year of listing to the deadline of the database).

As shown in Fig 6, it is easy to see that for the two companies with the same ranking, there are no particularly significant weaknesses in the selection of deviation indicators compared with the ratio indicators screened. Especially in asset size and cash flow. Then call back to Fig 7, although the assets and cash values of Muyuan Food Co., Ltd. appear to be very high, they are not very large from the perspective of industry deviation rankings. This can also be understood as the generally high value of total assets and cash in the industry. On the other hand, it also confirms the characteristic that the indicators we proposed can eliminate industry scale effects.

### Cox company survival analysis with MCP penalty term

In order to further examine the role of deviation indicators in practical application problems, and systematically compare our proposed indicator. In this section, we apply these metrics to the bankruptcy prediction (also called financial distress prediction) problem. We input all the indicators mentioned into the COX survival analysis model and select variables via the MCP (Minimum Cascade Penalty) method [45]. We can test what indicators will be filtered out.

Cox survival ratio analysis is one of the most popular survival analysis models, and it is also a common method to analyze the financial distress of companies [46, 47]. We conduct experiments on whether to use the deviation index. In many studies, it is found that the MCP method has good theoretical properties, and it can effectively improve the prediction accuracy of the model in the variable screening stage of Cox [48].

We used the data with variables (see Table 8) to train the COX+MCP model to predict whether the company will go bankrupt or not within three years. To verify the role of deviation indicators in the prediction of bankruptcy, we combine these indicators with all other available variables, including financial total volume indicators, financial ratio indicators, and selected macroeconomic variables together as the input features to predict bankruptcy and let the variable selection method MCP to select the useful variables automatically.

**Table 8:** Variables used.

Categories	Variables
Total volume data	C of SLS, EMPS, GR PRFT, NET INC, RET EARN, SALES
	1YR DBT, ASSETS, CASH, CMN SH, DVC, DVP
	LT DBT, MIN INT, PREF STK, TOT TAX, EBITDA, INT EXP
	R&D, SG&A, TOT LIA, Acquisitions-Income Contrib
	Acquisitions-Sales Contrib, BUS SEGS, Stores
	Store closings, Store openings
Deviation indicators	C of SLS_scale, LT DBT_scale, TOT LIA_scale, SG&A_scale, RET EARN_scale
	INT EXP_scale, R&D_scale, NET INC_scale, EBITDA_scale, GR PRFT_scale
Financial ratio	Cash ratio, Asset liability ratio, Ratio of short-term liabilities
	Ratio of operating cost in total sales, Ratio of cost of sales in total sales
	Profit ratio, Net operating rate, Interest Coverage
	Ratio of net income to total profit, Ratio of EBITDA to total profit
	Ratio of R & D investment
Macroeconomic variables	share, share_fine, gdp growth_rate, export_rate
	import_rate, fdirate, cpi, stock volatile

See Table 15 in S2 (S1 File) for full variable names.

The sampled data all come from the annual data of the 1950–2009 financial reports of North American listed companies in the COMPUSTAT dataset. The label of whether they are in financial distress is given by the “dlsrn” item in the dataset. For the companies marked as bankrupt, the data of any one of the three years before the bankruptcy is selected as the feature. After the screening, a total of 21,738 companies were selected. In the cross-validation experiment, we randomly selected 2,174 samples as the test set and the rest as the training samples. In addition to the 27 original financial indicators, 11 additional deviation indicators (use the number of employees as the proxy of size) and the corresponding ratio indicators, as well as six macroeconomic indicators, two market share indicators(‘share’ and ‘share_fine’ both describe the market share but via two calculation methods) are calculated (there are 57 indicators in total). The macroeconomic indicators and market share data come from the National Bureau of Statistics of the United States.

We use the package “ncvreg” to implement the COX+MCP method. The input data format is shown in Table 9.

**Table 9:** Sampled records of data for training.

Sample number		1	2
name		OMNI ENERGY SERVICES CORP	ACTON CORP -OLD
Total volume data (27)	C of SLS ($)	22380149.55	12944174.37
	…	…	…
	EMPS(person)	190	380
Financial ratio(11)	SG&A/SALES	1474944.497	-26852043.62
	…	…	…
	GR PRFT/SALES	33887154.55	32897038.76
Financial ratio(11)	SALES_s	-0.956	-0.907
	…	…	…
	SGA_s	-1.074	-0.050
Macroeconomic variables(8)	GDP growth	0.034	0.111
	…	…	…
	export rate	-0.054	0.059
**age**		15	27
bankrupt label		0	1

See Table 15 in S2 (S1 File) for full variable names.

The experimental steps are as follows:

1. Randomly divide the entire data set into the training set and the test set with 8:2;
2. Input data (training set, 57-dimensional feature+bankruptcy label+age) into the *ncvreg* package in *R* language and use COX with MCP to select the significant indicators(with hyper-parameter λ = 0.05);
3. Record the selected indicators;
4. Using these indicators as a set of new input variables, and call COX in the *ncvreg* package again;
5. Use the trained model to evaluate the test set and output AUC.
6. Repeat the above steps 1–4 ten times to obtain the results (to eliminate randomness).

Through this process, we get the variables selected in each iteration that may be discriminated due to the randomness of the method. We count the repeating times of each selected variable, and the results are collected in Table 10.

**Table 10:** Most related variables selected by COX+MCP method.

Variables	Times repeated
BUS SEGS, Store closings	10
share, share_fine	
TOT LIA_s, SG&A_s	
R&D_s, NET INC_s	
EBITDA_s, GR PRFT_s	
RET EARN_s	
GDP growth, fdirate, stocksd	
Ratio of EBITDA to total profit	2
Asset liability ratio	1
NET INC, RET EARN, CASH	

AUC_train = 0.707, AUC_test = 0.621, ‘_s’ represents the deviation index of the corresponding variable.

It can be seen that the deviation indicator appears repeatedly in ten cross-validation experiments, followed by the market share and some macroeconomic indicators appearing most frequently, and only two ratio indicators, namely the asset-liability ratio, and the ratio of EBITDA to total profit are selected. This proves that using the deviation indicators can provide more information for bankruptcy prediction. In fact, whether a company is bankrupt is not only related to its own development and the current market environment, but it is more related to its own situation and the relative quality of other companies in the market environment under competition. This information can be given by the deviation indicators. The deviation indicators not only contain the information of the ratio (because subtraction under logarithm is equivalent to division) but also contain higher information than ratio indices on the relative position within a market. Therefore, in the bankruptcy prediction problem, when there contain deviation indicators under the circumstances, the number of finalists for financial ratio indicators would be greatly reduced.

### Deviation analysis on the industrial level

The above perspectives are all from the perspective of enterprise evaluation, but scaling laws exist not only in individual industries but also in the general market (Fig 2a). In Section 3.5, we found that the deviation can not only be used for enterprise evaluation but also when we perform power-law fitting on the entire market to find the industry average deviation (Table 11), the industry average deviation at this time can reveal multi-dimensional industry characteristics and locate different trendy industries.

**Table 11:** The average deviation of each industry in 1990 (the beginning year of the dataset) uses the employee scale.

	SALES	GR PRFT	NET INC	ASSETS	TOT LIA	C of SLS	SG&A
Utilities	0.406^†^	0.317	0.548^†^	0.676	0.750	0.450^†^	-
Financials	0.334	0.317^†^	0.459	0.846^†^	0.922^†^	0.302	-
Energy	0.242	0.251	0.425	0.408	0.354	0.188	0.067
Consumer Staples	0.100	0.082	-0.087	-0.166	-0.167	0.098	0.176^†^
Materials	0.069	0.058	0.132	0.106	0.044	0.125	-0.067
Telecom -munication Services	-0.046	0.129	0.425	0.182	0.209	-0.144	0.176
Consumer Discretionary	-0.093	-0.125	-0.296*	-0.229	-0.207	-0.070	-0.043
Industrials	-0.100	-0.163	-0.243	-0.251	-0.252	-0.071	-0.113
Information Technology	-0.122	-0.053	-0.225	-0.320	-0.381	-0.194	0.085
Transportation	-0.141	-0.391	-0.230	-0.556*	-0.511*	-0.038	-0.770*
Health Care	-0.316*	-0.203*	-0.205	-0.334	-0.388	-0.280*	0.036
Unknown	-0.152	-0.175	-0.038	-0.234	-0.000	-0.146	0.023
MEAN	0.230	0.205	0.295	0.374	0.380	0.233	0.058

The notation † indicates the first industry in each attribute, while * indicates the last.

Through observation, it is found that the table can reflect the temporal characteristics. For example, in the financial industry is easy to form some companies with high capital scale and high debt. If the mean deviation is regarded as the development characteristics of various industries in that year, it can also be seen that several industries with rapid development in the 1990s are mainly Utilities, Financials, Energy, Consumer Staples, and other industries in the traditional industries of Finance and consumer goods; Similarly, when we count the deviation characteristics of various industries in later years like 2008, we find that the characteristics of these industries have changed, and the communication industry has replaced the daily consumer goods industry as one of the trendy industries. We can make a multi-temporal analysis through this way to find the change mode of industry characteristics during the period.

**Fig 8:**
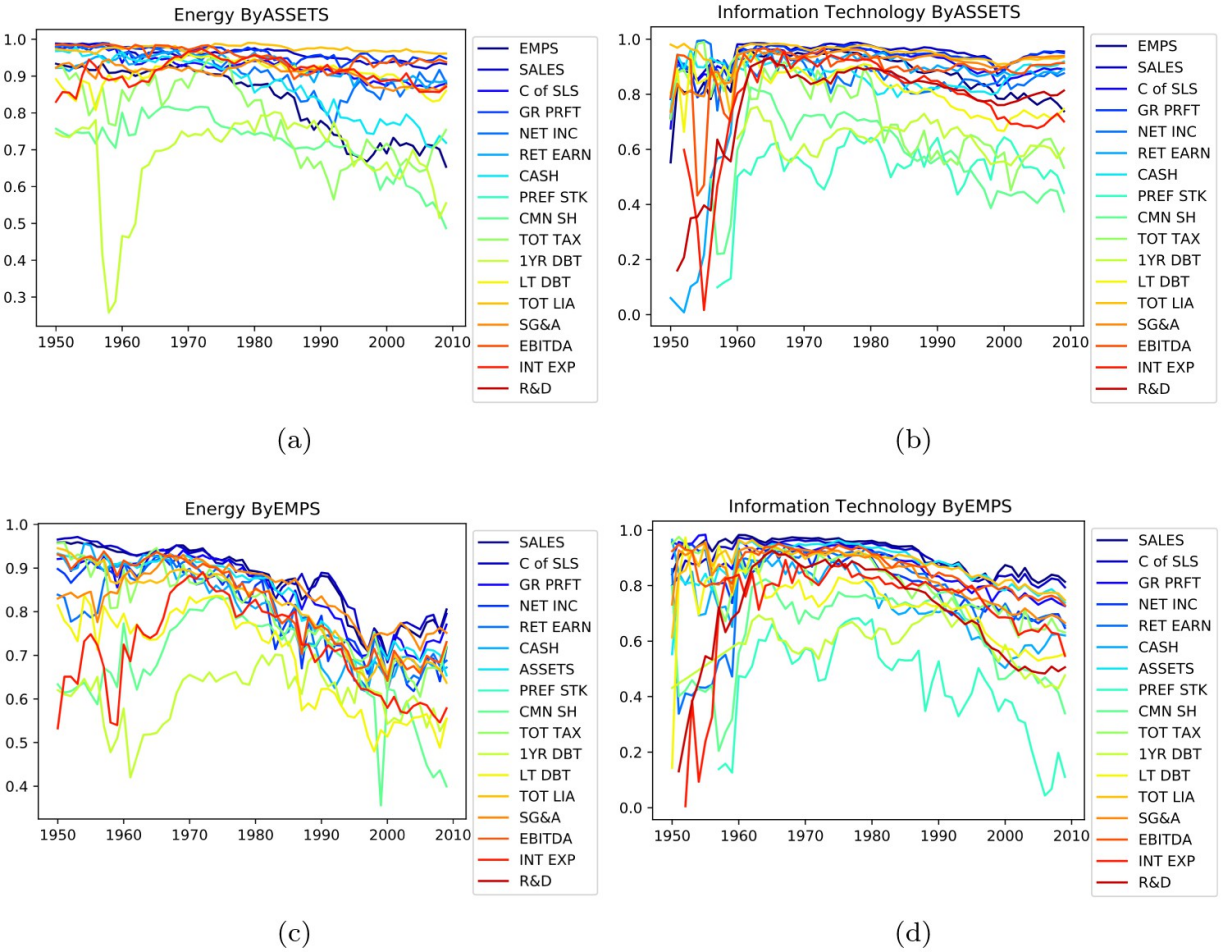
The change of the correlation coefficient between the number of employees and asset size in different indicators over time(taking the industry of Energy and IT as examples).

And then, when scoring the goodness of fit of financial indicators based on the number of employee size, it is found that the correlation coefficient between employee size and other indicators shows a significant downward trend over time (as shown in Fig 8), while there is no obvious time trend when using the asset scale to explain these indicators, which may be related to the adjustment of company industrial structure and the transformation from human-driven to technology-driven, Looking at the sub-industries, it is mainly reflected in the Energy, Materials, Industrials, and Information Technology.

## Conclusion and discussion

The existence of the scaling law of companies makes the conventional classic financial ratio index cannot evaluate company performance well. Thus, this paper proposes a new type of indicator based on the scaling law deviations. This indicator implies an industry background that can be used to evaluate the real development status of companies on the basis of eliminating the scaling law and facilitating the quantitative comparisons and ranking to assist in screening more potential individuals.

In the Cox survival analysis of company financial distress, we found that the frequency of deviation indicators which are selected by the repeating MCP variable selection process is much higher than the total volume indices and ratio indices. This can prove that deviation indicators can provide more information for predicting company’s financial distress.

Finally, we find that the scaling laws exist not only in individual industries but also in the entire market in general (Fig 2a). Also, we found that the deviation index can not only be used for enterprise evaluation but also can evaluate the whole industry.

In summary, the advantages of the deviation indicators are as follows:

1. The deviation index can reveal the general level of the company within an industry: its sign directly reflects the pros and cons of the company compared with the industry average, and the value directly reflects the size of the gap;
2. Since the deviation index is descaled, it is easy to filter out small companies which may have great potential. However, conventional methods based on total volume or financial ratio can not do because they are size-dependent;
3. The deviation index can provide more information on the financial health of companies, so as to assist in solving practical decision-making problems such as bankruptcy prediction;
4. The use of deviation indicators is more flexible: it can not only evaluate the financial status of companies qualitatively and quantitatively but also evaluate companies or industries from multi-dimensional and multi-industrial perspectives.

The deviation index is particularly suitable for quantitative evaluation of the financial and operating conditions of companies and can be effectively applied by specialized enterprise evaluation institutions to assist users in making informed decisions. It enables us to identify popular industries and companies with greater potential within those industries.

## Supporting information

S1 File (DOCX)
