# Supplementary material for: Company scaling and its deviations: New indicators for enterprise evaluation and bankruptcy prediction
Source: PLoS One. 2023 Oct 23;18(10):e0287105. doi: 10.1371/journal.pone.0287105 (PMC10593223; doi:10.1371/journal.pone.0287105)
Supplement: S1 File — (DOCX) [file pone.0287105.s001.docx]

Supporting information

# S1 Discussion about the robustness of scaling laws

We use the ordinary least square (OLS) method to fit the logarithmic power law relationship. It is necessary to test the hypothesis that the residual error conforms to a normal distribution to prove the robustness of the parameter estimation. Therefore, a QQ diagram of randomly selected variables is shown in Fig 9.


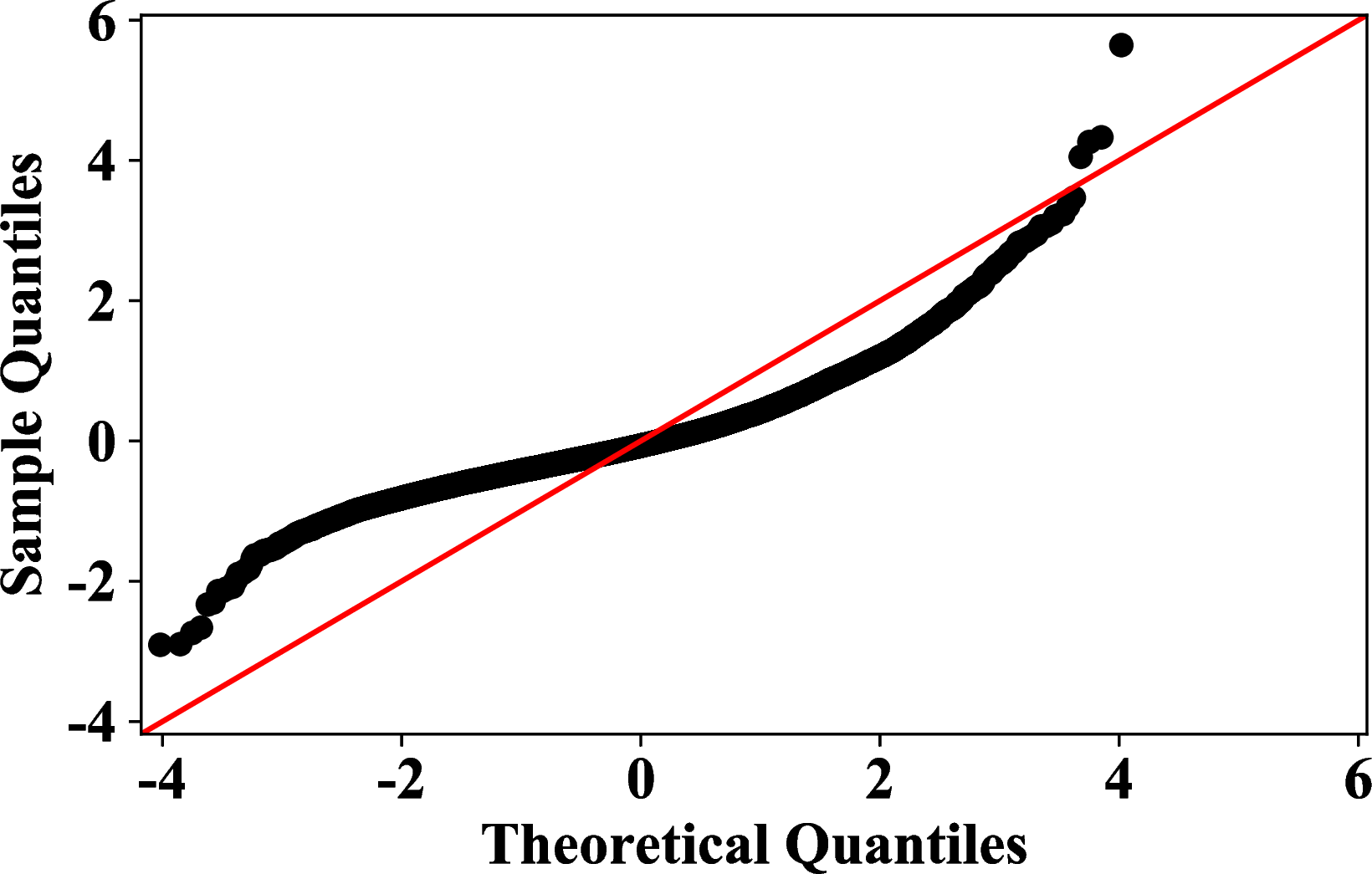

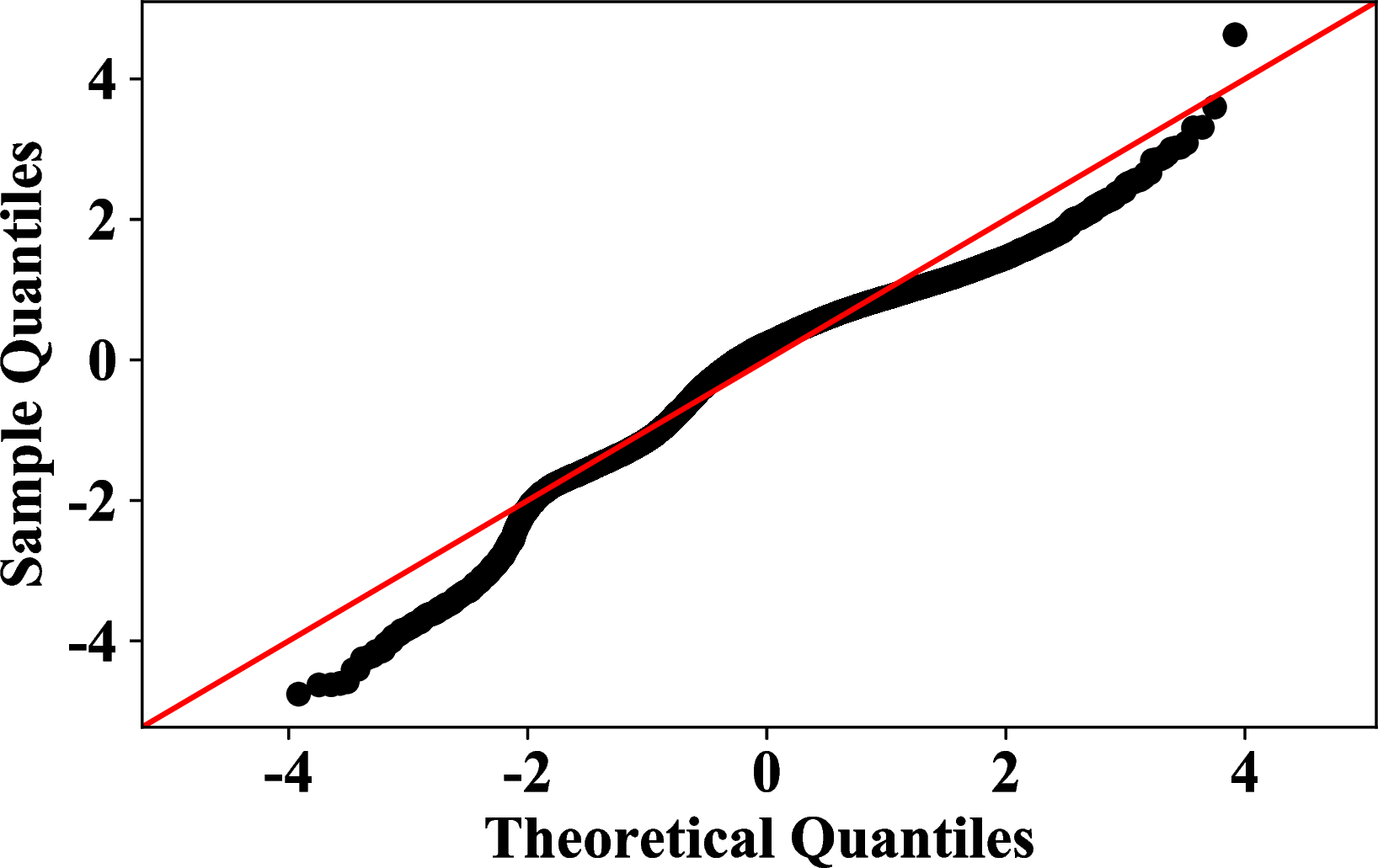


(a) (b)

**Fig 9. Fitting residual QQ diagram. Figure(a) on the left shows ASSETS and Figure(b) on the right shows EMPS. The scale variable is SALES. The red line is a 45° diagonal.**

It is obvious that the distribution of some residual points deviates significantly from the normal distribution. This requires the use of appropriate methods to correct their fitting results. It is necessary to test whether there is heteroscedasticity or autocorrelation in the data. Firstly, is heteroscedasticity, for which we use the White test method [1]. After fitting the model, we use the “bptest” function from the “lmtest” library by language R to perform White’s test to determine if heteroscedasticity is present. The results are shown in the following Table 12.

**Table 12. Results of White test. df means the degrees of freedom.**

| Variables | df | p-value |
| --- | --- | --- |
| EBITDA | 1 | <2.2e-16 |
| C of SLS | 1 | <2.2e-16 |
| GR PRFT | 1 | <2.2e-16 |
| NET INC | 1 | <2.2e-16 |
| RET EARN | 1 | <2.2e-16 |
| LT DBT | 1 | <2.2e-16 |
| ASSETS | 1 | <2.2e-16 |
| TOT LIA | 1 | <2.2e-16 |
| TOT TAX | 1 | <2.2e-16 |
| EMPS | 1 | 5.99E-15 |
| INT EXP | 1 | <2.2e-16 |
| CASH | 1 | <2.2e-16 |
| SG&A | 1 | <2.2e-16 |
| 1YR DBT | 1 | <2.2e-16 |
| DVC | 1 | <2.2e-16 |
| R&D | 1 | <2.2e-16 |
| CMN SH | 1 | 0.262 |

Data here we select companies from North America in the year 2018 for example and as a correct result for Table 1. All variables have a very low p-value and the degree of freedom is 1. This proves the existence of heteroscedasticity in the data. Next is the Durbin-Watson test [2] for autocorrelation. We also use the method “dwtest” by language R to conduct this test on all the variables. Results are as follows in Table 13.

**Table 13. Results of Durbin-Watson test. DW means the statistic value.**

| Variables | DW | p-value |
| --- | --- | --- |
| EBITDA | 1.9193 | 1.22E-13 |
| C of SLS | 1.9433 | 2.17E-07 |
| GR PRFT | 1.9377 | 2.56E-07 |
| NET INC | 1.8744 | 1.48E-07 |
| RET EARN | 1.759 | <2.2e-16 |
| LT DBT | 1.8126 | <2.2e-16 |
| ASSETS | 1.7463 | <2.2e-16 |
| TOT LIA | 1.7396 | <2.2e-16 |
| TOT TAX | 1.9611 | 0.000622 |
| EMPS | 1.3118 | <2.2e-16 |
| INT EXP | 1.7566 | <2.2e-16 |
| CASH | 1.7164 | <2.2e-16 |
| SG&A | 1.647 | <2.2e-16 |
| 1YR DBT | 1.9232 | 1.81E-08 |
| DVC | 1.6299 | <2.2e-16 |
| R&D | 1.6143 | <2.2e-16 |
| CMN SH | 1.8797 | <2.2e-16 |

All of the values of P tend to be small. It proved that all variables listed have an autocorrelation relationship. At this point, we can determine that our data has both heteroscedasticity and autocorrelation. Then we use the Newey-West estimator to correct the fitting coefficients. Newey-West in R can be accomplished by the method “NeweyWest”. Finally, we can give the slope and intercept of the modified linear fitting as shown in Table 14. Results here can be seen as correct for Table 1.

**Table 14. Corrected results of OLS. The slope and intercept represent *β* and lg*a* in Eq.2 respectively.**

|  |  | coefficient | std err | p-value |
| --- | --- | --- | --- | --- |
| EBITDA | slope | 0.76[0.74, 0.78] | 0.012 | <2.2e-16 |
|  | intercept | -0.52[-0.57,-0.47] | 0.025 | < 2.2e-16 |
| C of SLS | slope | 0.96[0.95, 0.97] | 0.012 | <2.2e-16 |
|  | intercept | -0.13[-0.15,-0.11] | 0.011 | < 2.2e-16 |
| GR PRFT | slope | 0.84[0.82, 0.85] | 0.007 | <2.2e-16 |
|  | intercept | -0.27[-0.29,-0.23] | 0.016 | < 2.2e-16 |
| NET INC | slope | 0.69[0.65, 0.73] | 0.019 | <2.2e-16 |
|  | intercept | -0.95[-1.03,-0.86] | 0.043 | < 2.2e-16 |
| RET EARN | slope | 0.78[0.75, 0.81] | 0.015 | <2.2e-16 |
|  | intercept | -0.81[-0.87,-0.74] | 0.033 | < 2.2e-16 |
| LT DBT | slope | 0.93[0.90, 0.95] | 0.015 | <2.2e-16 |
|  | intercept | -0.72[-0.77,-0.67] | 0.011 | < 2.2e-16 |
| ASSETS | slope | 0.77[0.76, 0.78] | 0.007 | <2.2e-16 |
|  | intercept | 0.75[0.72,0.78] | 0.015 | < 2.2e-16 |
| TOT LIA | slope | 0.84[0.82, 0.85] | 0.007 | <2.2e-16 |
|  | intercept | 0.23[0.20,0.26] | 0.016 | < 2.2e-16 |
| TOT TAX | slope | 0.64[0.62, 0.66] | 0.009 | <2.2e-16 |
|  | intercept | -1.07[-1.061,-0.97] | 0.022 | < 2.2e-16 |
| EMPS | slope | 0.81[0.78, 0.84] | 0.014 | <2.2e-16 |
|  | intercept | -2.72[-2.80,-2.63] | 0.042 | < 2.2e-16 |
| INT EXP | slope | 0.80[0.79, 0.82] | 0.009 | <2.2e-16 |
|  | intercept | -1.58[-1.63,-1.53] | 0.023 | < 2.2e-16 |
| CASH | slope | 0.77[0.75, 0.79] | 0.009 | <2.2e-16 |
|  | intercept | -0.44[-0.50,-0.39] | 0.028 | < 2.2e-16 |
| SG&A | slope | 0.74[0.72, 0.76] | 0.009 | <2.2e-16 |
|  | intercept | -0.22[-0.26,-0.17] | 0.023 | < 2.2e-16 |
| 1YR DBT | slope | 0.74[0.73, 0.76] | 0.008 | <2.2e-16 |
|  | intercept | -0.96[-1.01,-0.91] | 0.023 | < 2.2e-16 |
| DVC | slope | 0.84[0.81, 0.86] | 0.011 | <2.2e-16 |
|  | intercept | -1.21[-1.28,-1.14] | 0.034 | < 2.2e-16 |
| R&D | slope | 0.56[0.53, 0.59] | 0.017 | <2.2e-16 |
|  | intercept | -0.68[-0.77,-0.58] | 0.048 | < 2.2e-16 |
| CMN SH | slope | 0.69[0.67, 0.70] | 0.007 | <2.2e-16 |
|  | intercept | 0.44[0.41,0.48] | 0.016 | < 2.2e-16 |

Here, the correction is only applied to the upper and lower bounds of the variance of the parameter estimation. The parameters themselves are reasonable as long as they fall within the error range. After inspection, the parameter fitting results given in Table 1 are basically within the error range.

# S2 Other Tables and figures

**Table 15. The abbreviations of financial variables use in this article correspond to their meanings.**

| Abbreviation | Full name | Description |
| --- | --- | --- |
| DLRSN | Reason for deletion | Codes for why a company stopped reporting sales. |
| EMPS | Employees | - |
| SALES | Total sales | - |
| C of SLS | Cost of sales | Costs to the company of the goods they sell. |
| GR PRFT | Gross profits | Total sales - cost of sales. |
| NET INC | Net income | Total sales - all other expenses, split into dividend payouts and retained earnings. |
| RET EARN | Retained earnings | Profits funneled back into the company, Profits - total dividends (common + preferred). |
| CASH | Cash on hand | Cash on hand. |
| ASSETS | Total assets | Anything a company owns that has value. |
| MIN INT | Minority interest | The portion of a subsidiary corporation's stock that is not owned by the parent corporation. |
| PREF STK | Prefered stock | Special equity security that has properties of both an equity and a debt instrument. |
| CMN SH | Common shareholders | The number of individuals that own stock in a company. |
| TOT TAX | Total income taxes | Total income taxes. |
| 1YR DBT | Debt due in 1 year | Debt due in 1 year. |
| LT DBT | Long-term debt | Debt due over 1 year. |
| DVC | Common dividends | Cash paid to common stock holders. |
| DVP | Preferred dividends | Cash paid to preferred stock holders. |
| TOT LIA | Total liabilities | All other income and costs other than operating costs. |
| SG&A | Selling and general administrative expense | Operating costs (in addition to R&D). |
| EBITDA | Earnings before income, taxes, depreciation and amortization | Gross profits - SG&A, cash earning before reductions. |
| INT EXP | Interest expense | Cost of borrowing money. |
| R&D | Research and development | Research and development expenses |
| BUS SEGS | Business segments | Business of independent segments. |
| Stores | Stores | Number of establishments. |
| AQI | Acquisitions-Income Contrib | Contribution of acquisitions to income. |
| AQS | Acquisitions-Sales Contrib | Contribution of acquisitions to sales. |
| share | Market share | Market share. |
| share_fine | Market share | Market share (calculated in another way). |
| gdp growth_rate | GDP growth rate | GDP growth rate. |
| export_rate | Export rate | Export rate. |
| import_rate | Import rate | Import rate. |
| fdirate | Foreign Direct Investment Rate | A macro-financial indicator, foreign direct investment, annual growth rate (year-on-year). |
| cpi | Consumer Price Index | An indicator of price changes that reflects the prices of products and services related to residents' lives. |
| stock volatile | Stock market volatility | The volatility of the stock market, which is calculated based on the data of a certain stock market in the United States. |

**Table 16. Equation variable description.**

| Variable | Description |
| --- | --- |
| *ζ* | Deviation index |
| *X* | Variable of company size. |
| *Y* | Other financial variables. |
| $\hat{Y}$ | The result of scaling law fitting. |
| *α* | Normalizing coefficient of scaling. |
| *β* | Scaling exponent. |
| *r* | Variable ranking. |

**Table 17. Scaling law between SALES and other variables of the Chinese market in 2020 (sorted by scaling exponent *β*). Here we also calculate the value of *P*. However, the table does not specify because all variables are calculated to approximate 0.00.**

| Variables | Scaling Coefficient *α* | Scaling Exponent *β* | Standard Deviation of *β* | *R*^2^ | Number of samples |
| --- | --- | --- | --- | --- | --- |
| SG&A | 0.28 | 1.04 | 0.007 | 0.94 | 1654 |
| TOT LIA | 2.88 | 0.93 | 0.013 | 0.74 | 1658 |
| EBITDA | 0.68 | 0.90 | 0.018 | 0.65 | 1357 |
| PRE PRFT | 0.98 | 0.86 | 0.020 | 0.58 | 1343 |
| TOT TAX | 0.21 | 0.82 | 0.012 | 0.73 | 1657 |
| CASH | 10.72 | 0.81 | 0.015 | 0.64 | 1658 |
| ASSETS | 0.65 | 0.78 | 0.009 | 0.81 | 1658 |
| TOT TAX | 147.91 | 0.78 | 0.009 | 0.81 | 1658 |
| RET EARN | 32.36 | 0.76 | 0.017 | 0.59 | 1362 |
| C of SLS | 26.30 | 0.67 | 0.022 | 0.35 | 1653 |
| R&D | 33.88 | 0.64 | 0.020 | 0.39 | 1652 |

# New References

[1] White H. A Heteroskedasticity-Consistent Covariance Matrix Estimator and a Direct Test for Heteroskedasticity. Econometrica: journal of the Econometric Society, 1980: 817-838.

[2] Durbin J, Watson G S. Testing for serial correlation in least squares regression. III. Biometrika, 1971, 58(1): 1-19.
